# Vascular dysfunction and fibrosis in stroke-prone spontaneously hypertensive rats: The aldosterone-mineralocorticoid receptor-Nox1 axis

**DOI:** 10.1016/j.lfs.2017.05.002

**Published:** 2017-06-15

**Authors:** Adam P. Harvey, Augusto C. Montezano, Katie Y. Hood, Rheure A. Lopes, Francisco Rios, Graziela Ceravolo, Delyth Graham, Rhian M. Touyz

**Affiliations:** a Institute of Cardiovascular and Medical Sciences, University of Glasgow, United Kingdom; b Department of Physiological Sciences, State University of Londrina, Londrina, Brazil

**Keywords:** Oxidative stress, Vascular remodeling, Vascular function, Signal transduction, Hypertension, Mineralocorticoid receptors

## Abstract

**Aims:**

We questioned whether aldosterone and oxidative stress play a role in vascular damage in severe hypertension and investigated the role of Nox1 in this process.

**Materials and methods:**

We studied mesenteric arteries, aortas and vascular smooth muscle cells (VSMC) from WKY and SHRSP rats. Vascular effects of eplerenone or canrenoic acid (CA) (mineralocorticoid receptor (MR) blockers), ML171 (Nox1 inhibitor) and EHT1864 (Rac1/2 inhibitor) were assessed. Nox1-knockout mice were also studied. Vessels and VSMCs were probed for Noxs, reactive oxygen species (ROS) and pro-fibrotic/inflammatory signaling.

**Key findings:**

Blood pressure and plasma levels of aldosterone and galectin-3 were increased in SHRSP versus WKY. Acetylcholine-induced vasorelaxation was decreased (61% vs 115%) and phenylephrine-induced contraction increased in SHRSP versus WKY (E_max_ 132.8% vs 96.9%, *p* < 0.05). Eplerenone, ML171 and EHT1864 attenuated hypercontractility in SHRSP. Vascular expression of collagen, fibronectin, TGFβ, MCP-1, RANTES, MMP2, MMP9 and p66Shc was increased in SHRSP versus WKY. These changes were associated with increased ROS generation, 3-nitrotyrosine expression and Nox1 upregulation. Activation of vascular p66Shc and increased expression of Nox1 and collagen I were prevented by CA in SHRSP. Nox1 expression was increased in aldosterone-stimulated WKY VSMCs, an effect that was amplified in SHRSP VSMCs (5.2vs9.9 fold-increase). ML171 prevented aldosterone-induced VSMC Nox1-ROS production. Aldosterone increased vascular expression of fibronectin and PAI-1 in wild-type mice but not in Nox1-knockout mice.

**Significance:**

Our findings suggest that aldosterone, which is increased in SHRSP, induces vascular damage through MR-Nox1-p66Shc-mediated processes that modulate pro-fibrotic and pro-inflammatory signaling pathways.

## 1 Introduction

Hypertension-associated vascular damage is characterised by functional, structural and mechanical alterations comprising hypercontractility, endothelial dysfunction, inflammation, calcification and fibrosis [1], [2]. Molecular mechanisms underlying vascular changes in hypertension are incompletely understood, but a role for the mineralocorticoid hormone aldosterone, has been suggested. Aldosterone acts through genomic and non-genomic pathways to regulate blood pressure and electrolytic homeostasis. Accumulating evidence suggests that aldosterone also has direct vascular effects and that it is a potent pro-fibrotic agent in cardiovascular remodeling in hypertension [3]. Evidence from animal models and clinical trials in patients with heart failure demonstrate that blockade of the mineralocorticoid receptor (MR) through which aldosterone signals, is cardio- and vaso-protective [4], [5]. In the context of vascular function, a role for aldosterone-MR signaling remains controversial because aldosterone has been shown to induce both vasorelaxation and vasoconstriction [6]. Recently, a role for reactive oxygen species (ROS) has been suggested to mediate the detrimental effects of aldosterone in the vasculature [3].

Oxidative stress has been strongly implicated in many of the molecular processes associated with endothelial dysfunction, structural remodeling and vascular inflammation in hypertension [7], [8], [9]. In the vascular wall, NADPH oxidases (Noxs) are the predominant source of ROS and are upregulated in hypertension [7], [8], [9]. Nox isoforms 1,2,4 and 5 are expressed in human vascular tissue and appear to be dysregulated in pathological conditions [8], [9], [10]. Increased activation of Nox1, Nox2 and Nox5 has been demonstrated in hypertension-associated cardiovascular damage, whereas Nox4 activation has been associated with both beneficial [11], [12] and injurious effects [13].

A key role for Nox1 in redox signaling in angiotensin II (AngII)-dependent models of hypertension has been demonstrated [14]. Nox1 has also been shown to be linked to AngII and aldosterone signaling in vascular cells [15], [16], [17]. However, the relationship between aldosterone and Nox1 in the context of vascular remodeling in hypertension is incompletely defined.

The stroke-prone spontaneously hypertensive rat (SHRSP) represents a robust model of human hypertension with end-organ damage. In this model, upregulation of Nox1 has been observed [18] and inhibition of aldosterone signaling is associated with beneficial results [19]. It has yet to be determined whether aldosterone mediates its effects through activation of Noxs. Here we hypothesise that in SHRSP rats, aldosterone promotes vascular damage through mechanisms involving Nox1.

## 2 Methods

### 2.1 Experimental animals

Animal experiments were performed in accordance with the United Kingdom Animals Scientific Procedures Act 1986 and ARRIVE Guidelines [20] and approved by the institutional ethics review committee. Male WKY and SHRSP rats aged at 16–18 weeks were used for experimentation. In additional studies rats were treated with canrenoic acid (CA) (10 mg/kg/day, in drinking water) from weaning until 18 weeks old. Male Nox1 knockout mice (Nox1-/y) aged 10–12 weeks were infused with aldosterone (3 × 10^− 4^ mol/L/Kg/day) for 4 weeks by osmotic minipumps (model 2004, Alzet, Cupertino, CA) implanted under isoflurane (3% induction; 1.5% maintenance) anaesthesia. Animals were euthanized by exsanguination following cardiac puncture with immediate dissection of tissues that were rinsed, snap-frozen in liquid nitrogen and stored at − 80 °C or fixed in formalin for preparation of histological analysis.

### 2.2 Blood pressure measurement

Mean arterial pressure was measured in WKY and SHRSP rats using tail-cuff plethysmography. Rats were placed in isolation chambers on a heated platform, and blood pressure measurements were obtained. Before recording measurements, animals were trained for 5 consecutive days. Animals were initially subjected to preliminary measurements, which were not recorded; followed by 10 recorded measurements. Mean arterial pressure is displayed as the average of 10 measurements.

### 2.3 Plasma aldosterone, plasma galectin-3 and vascular nitrotyrosine measurements by ELISA

Plasma aldosterone (Cayman Chemical, Ann Arbor, USA), plasma galectin-3 (Life Technologies, Paisley, UK) and mesenteric artery nitrotyrosine (Abcam, Cambridge, UK) levels were measured by ELISA according to manufacturer's instructions.

### 2.4 Vascular smooth muscle cell isolation

Rat mesenteric arteries underwent enzymatic digestion for culture of vascular smooth muscle cells (VSMCs) as previously described [21]. Experiments were performed on low-passage cells (passage 4–7). Prior to experimentation, cell cultures were rendered quiescent by serum deprivation (0.5% FBS) for 18 h.

### 2.5 Vascular structure and function in mesenteric arteries

Rat mesenteric arteries were cut into 2 mm ring segments and mounted on isometric wire myographs (Danish Myo Technology, Denmark) filled with 5 mL of physiological saline solution and continuously gassed with a mixture of 95% O_2_ and 5% CO_2_ while maintained at a constant temperature of 37 ± 0.5 °C. Following 60 min of equilibration, the contractility of arterial segments was assessed by the addition of 12 × 10^− 2^ mol/L KCl solution. The integrity of the endothelium was verified by relaxation induced by acetylcholine (Ach; 10^− 5^ mol/L) in arteries pre-contracted with phenylephrine (PE; 10^− 6^ mol/L). In additional experiments vessels were preincubated for 60 min prior to the curves with the mineralocorticoid receptor blocker, eplerenone (10^− 6^ mol/L), Rac1/2 inhibitor, EHT1864 (10^− 5^ mol/L) and Nox1 inhibitor, ML171 (10^− 5^ mol/L).

Additional segments of mesenteric artery (~ 4 mm long) were studied as pressurized preparations using a Pressure Myograph System (110P system; Danish Myo Technology, Denmark) in Ca^2 +^ free physiological saline solution where intramural pressure was set to 70 mmHg for 30 min to allow vessels to equilibrate and generate a variable degree of myogenic tone. Vessels were subjected to an increasing intramural pressure gradient ranging from 10 to 120 mmHg at 5 min intervals. Inner and outer vessel wall measurements were recorded and used to calculate structural elastic properties. Statistical significance was assessed by non-linear regression analysis.

### 2.6 Collagen content assessed by picrosirius red staining

To assess vascular collagen expression, portions of abdominal aorta were fixed in 10% neutral buffered formalin following excision and paraffin embedding, where 5 μm sections were stained with picrosirius red (0.1% *w*/*v*). In order to distinguish between collagen type I and type III, stained sections were visualised and imaged under polarised light using an Olympus BH-2 microscope (Olympus, Japan). Data were quantified by digital image analysis (ImageJ) with the observer blinded to sample identity and expressed as the ratio of collagen type I to collagen type III.

### 2.7 Immunoblotting

Protein was extracted from cleaned and snap frozen mesenteric arteries and aortas from WKY, SHRSP rats, and from cultured rat VSMCs. Protein (30 μg) was separated by electrophoresis on a polyacrylamide gel and transferred to a nitrocellulose membrane. Non-specific binding sites were blocked with 5% bovine serum albumin in Tris-buffered saline (TBS) solution. Membranes were then incubated with specific antibodies overnight at 4 °C. Membranes were washed 3 times with TBS-Tween 20 and incubated with specific secondary antibodies for 1 h at room temperature. Signals were revealed after reaction with enhanced chemiluminescence. Results were normalised to α-tubulin or β-actin, as indicated in the figures and are expressed as arbitrary units. In most of our studies we used α-tubulin as the housekeeping protein, except for the studies assessing p66Shc expression in aorta, where we used β-actin as our internal control. This related to technical aspects, where β-actin detection was superior to that of α-tubulin. Antibodies were as follows: anti-TGFβ (SC146, Santa Cruz, USA) anti-fibronectin (F3648, Sigma-Aldrich, UK), anti-phospho-p66Shc (566,807, Calbiochem, USA), anti-α-tubulin (AB4074, Abcam, UK), anti-phospho-p38MAPK (9211S, Cell Signaling, UK) anti-total-p38MAPK (9212S, Cell Signaling, UK), Nox1 (sc-25,545, Santa Cruz, USA) and anti-β-actin (ab8229, AbCam, UK).

### 2.8 Lucigenin-enhanced chemiluminescence

Lucigenin-enhanced chemiluminescence was used to assess ROS production in rat mesenteric arteries and VSMCs stimulated with aldosterone (10^− 7^ mol/L, 30 min; Sigma, UK). In some experiments, cells were pre-incubated for 30 min with ML171 (10^− 6^ mol/L; Tocris, UK). After stimulation, cells were rinsed with ice-cold PBS and harvested in ROS phosphate buffer (5 × 10^− 2^ mol/L KH_2_PO_4_, 10^− 2^ mol/L EGTA, 1.5 × 10^− 1^ mol/L Sucrose). NADPH (10^− 4^ mol/L) was added to the suspension containing lucigenin (5 × 10^− 6^ mol/L). Luminescence was measured before and after addition of NADPH. A buffer blank was subtracted from each reading. Results are expressed as a fold change in relative light units (RLU) per microgram of protein, as measured by the DC assay (Bio-Rad, UK).

### 2.9 Quantitative real-time polymerase chain reaction (PCR)

Quantitative real-time Polymerase Chain Reaction (Applied Biosystems) was used to analyze mRNA expression in rat mesenteric arteries; data are expressed as target gene/18 s housekeeping gene. Transcript expression in rat VSMCs was normalised to GAPDH, using Qiagen QuantiTech primer assays (Qiagen, UK). Relative gene expression was calculated by 2^ΔΔCt^ method, and the results were reported as arbitrary units relative to the control conditions. Primers were designed using Primer3 software with sequences as shown in Table 1.

**Table 1:** Primers targeted to rat genes for qRT-PCR analysis.

Rat gene	Forward primer	Reverse primer
Nox1	TCCCTTTGCTTCCTTCTTGA	CCAGCCAGTGAGGAAGAGTC
NoxA1	TTACTGTGCCCCTGAAGGTC	CTCGGGCTTTGTAGCTGAAC
NoxO1	TCCAGACGTTTGCCTTCTCT	CGTGTCAACAATGGAGCATC
Nox2	ACCCTTTCACCCTGACCTCT	TCCCAGCTCCCACTAACATC
Nox4	CCAGAATGAGGATCCCAGAA	AGCAGCAGCAGCATGTAGAA
P22phox	TTGTTGCAGGAGTGCTCATC	CAGGGACAGCAGTAAGTGGA
P47phox	AGCTCCCAGGTGGTATGATG	ATCTTTGGCCGTCAGGTATG
MMP2	AGCTCCCGGAAAAGATTGAT	TCCAGTTAAAGGCAGCGTCT
MMP9	CACTGTAACTGGGGGCAACT	CACTTCTTGTCAGCGTCGAA
MCP-1	CAGTTAATGCCCCACTCACC	TTCCTTATTGGGGTCAGCAC
RANTES	ATATGGCTCGGACACCACTC	CCACTTCTTCTCTGGGTTGG
18S	AAGTCCCTGCCGTTTGTACACA	GATCCGAGGGCCTCACTAAAC

### 2.10 Measurement of vascular hydrogen peroxide (H_2_O_2_) production

The production of H_2_O_2_ in vascular tissue homogenate and VSMC lysate was measured using the horseradish peroxidase-linked Amplex Red™ fluorometric assay (Life Technologies, Paisley, UK) according to manufacturer's instructions.

### 2.11 Statistical analysis

All data are expressed as mean ± SEM unless otherwise indicated. Statistical comparisons of parameters between groups were made by student's *t*-test, 1- way and 2-way ANOVA followed by Bonferroni post-hoc tests as appropriate. *P* < 0.05 was considered statistically significant. Repeated measures ANOVA was used for comparison of groups within vascular reactivity studies. Data analysis was conducted using GraphPad Prism 5.0 (GraphPad Software Inc., San Diego, CA).

## 3 Results

### 3.1 Elevated blood pressure is associated with increased plasma aldosterone and galectin-3 levels in SHRSP rats

SHRSP rats had lower body weight (279.2 ± 9.8 g vs 353.5 ± 4.1 g, *P* < 0.001) and increased systolic blood pressure (183.8 ± 5.2 mm Hg vs 123.5 ± 2.1 mm Hg *P* < 0.001) compared to WKY rats. Analysis of plasma revealed elevated levels of aldosterone (99.5 ± 19.2 pg/mL vs 20.8 ± 5.3 pg/mL, *P* < 0.05) and the pro-fibrotic marker galectin-3 (233.8 ± 23.5 pg/mL vs 153.7 ± 25.1 pg/mL, *P* < 0.05) in SHRSP rats versus WKY rats. CA did not reduce blood pressure in either WKY (121.8 ± 11.02 mm Hg vs CA: 136.1 ± 10.32 mm Hg) or SHRSP (187.4 ± 7.53 mm Hg vs CA: 208.6 ± 3.76 mm Hg).

### 3.2 Vascular function and mechanics are altered in SHRSP rats

**Fig. 1:**
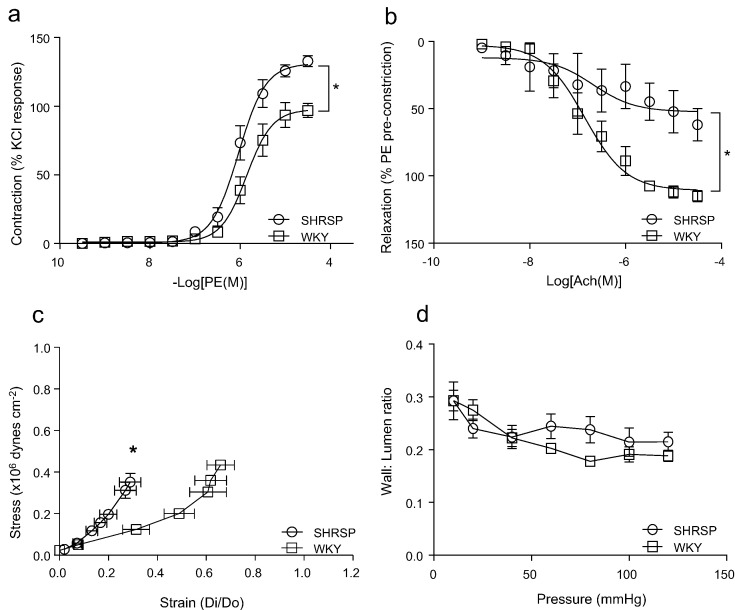
Assessment of vascular contraction to phenylephrine (PE) (a) and endothelial-dependent relaxation to acetylcholine (Ach) assessed by wire myography (b). Vascular stress-strain curve (c) and wall to lumen ratio (d) assessed by pressure myography in WKY and SHRSP rats. Curves represent the mean ± SEM (*n* = 8–15 per group). *P < 0.05 vs WKY.

Rat mesenteric arteries were studied by wire and pressure myography to assess endothelial function, vascular contractility and structural and mechanical properties. Hypertensive animals displayed altered vascular function characterised by hypercontractility and, increased sensitivity and maximal contractile response to phenylephrine (Fig. 1a). As previously demonstrated [21], [22], [23], endothelium-dependent vasorelaxation to acetylcholine was impaired in SHRSP rats (Fig. 1b). Endothelium-independent vasorelaxation was not altered in SHRSP rats (Supplemental Fig. 1). Vascular stiffness was increased in SHRSP rats as demonstrated by a leftward shift in the stress-strain curve (Fig. 1c), confirming previous findings [22]. Vascular media-lumen ratio and cross sectional area were not significantly modified in SHRSP versus WKY rats (Fig. 1d, Supplemental Fig. 2).

**Fig. 2:**
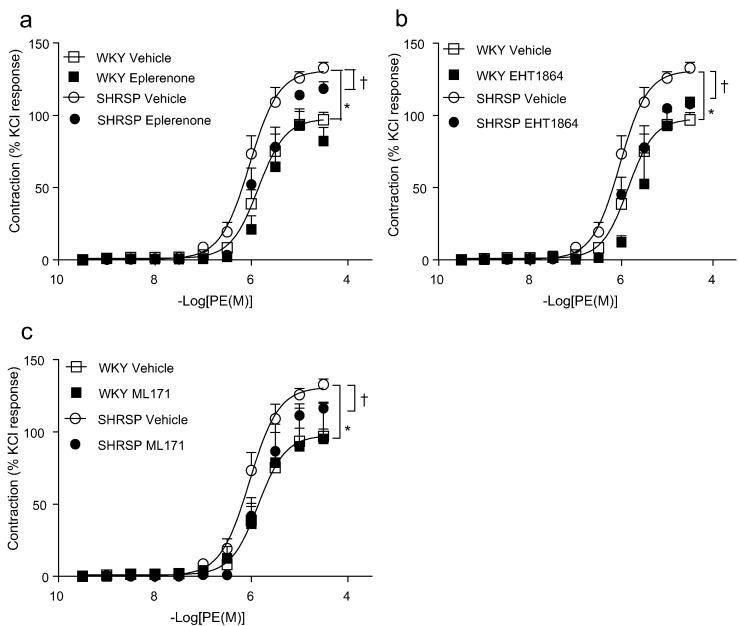
Effects of MR blockade and Rac and Nox inhibition on hypertension-associated vascular hypercontractility. Assessment of vascular contraction in rat mesenteric artery to phenylephrine (PE) in the presence of MR blocker, eplerenone (a), Rac1/2 inhibitor, EHT1864 (b) and Nox1 inhibitor, ML171 (c) assessed by wire myography, curves represent the mean ± SEM (*n* = 5–15 per group), *P < 0.05 WKY vehicle vs SHRSP vehicle curve; †*p* < 0.05 SHRSP vehicle vs SHRSP treated curve.

### 3.3 Vascular hypercontractility involves MR, Nox1 and Rac1/2 in SHRSP rats

To assess whether vascular dysfunction in SHRSP is related to MR-mediated effects, vessels were pre-incubated with the MR antagonist, eplerenone, which reduced the hypercontractile response to phenylephrine in SHRSP rats (Fig. 2a). Rac is an established cytosolic regulator of Nox activity. The involvement of Nox and Rac1/2 in hypertension-associated hypercontractility was assessed by pre-incubating vessels with the Rac1/2 inhibitor, EHT1864 (Fig. 2b) or the Nox1 inhibitor, ML171 (Fig. 2c). Both EHT1864 and ML171 attenuated hypercontractility in SHRSP vessels.

### 3.4 Vascular extracellular matrix remodeling and inflammatory marker expression are increased in SHRSP rats

**Fig. 3:**
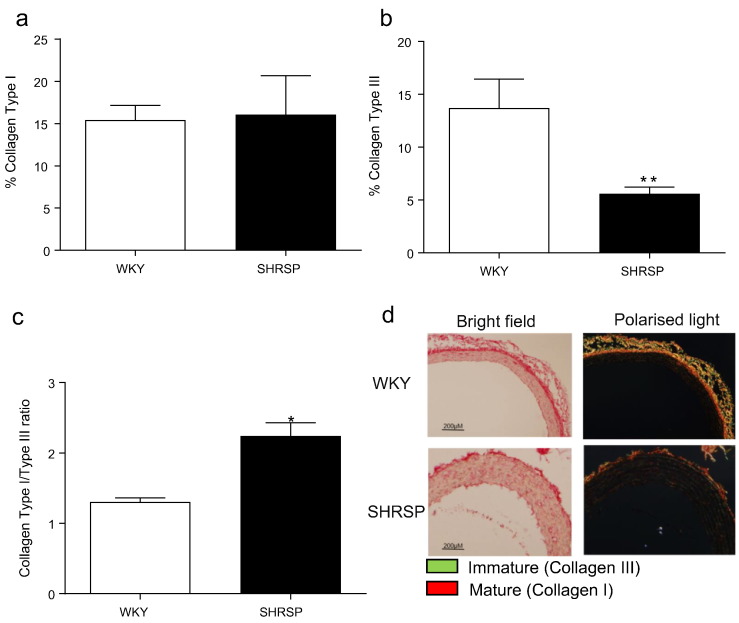
Vascular collagen expression in WKY and SHRSP rats. Mean quantification by Image J analysis of percentage of collagen type I (a), collagen type III (b), collagen type I to collagen type III ratio (c) assessed by picrosirius red staining analysed under polarised light. Representative images of WKY and SHRSP vessels stained with picrosirius red under bright-field and polarised light microscopy (d). Scale bar (−) represents 200 μM. Bars represent the mean ± SEM (*n* = 4–6 per group). *P < 0.05, **P < 0.001 vs WKY.

**Fig. 4:**
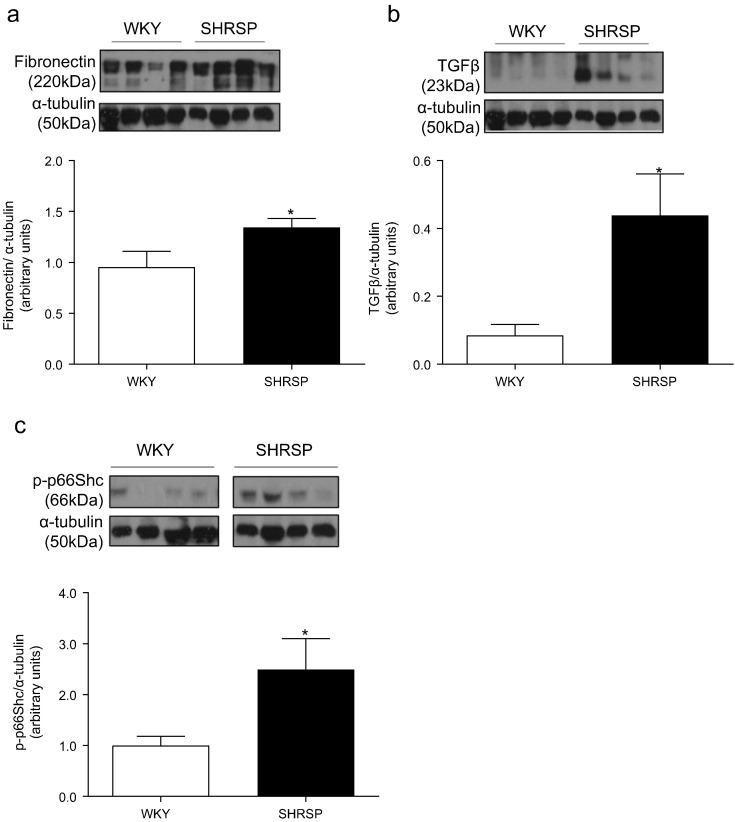
Vascular expression of matrix and pro-fibrotic signaling proteins in normotensive and hypertensive rats. Mesenteric artery protein expression of fibronectin (a) TGFβ (b) and phosphorylated p66Shc (c). Data are expressed as mean ± SEM, corrected to α-tubulin expression (*n* = 8–9 per group). *P < 0.05 vs WKY.

**Fig. 5:**
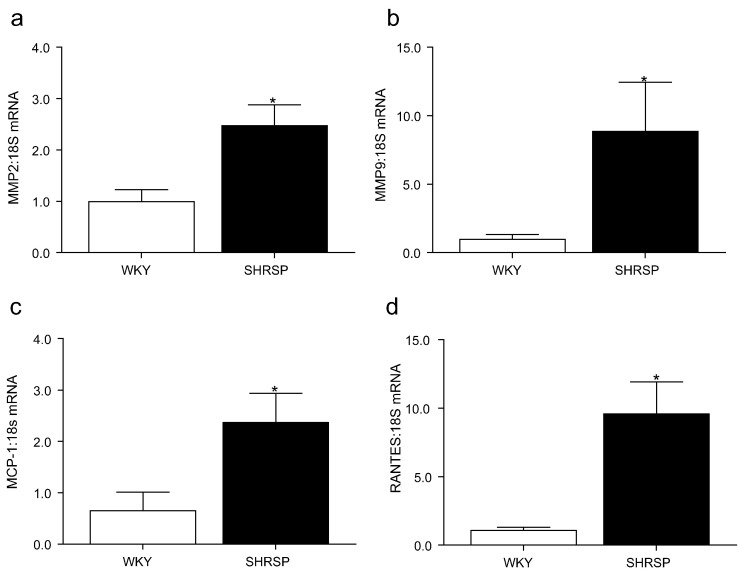
Vascular expression of matrix metalloproteinases (MMP) and pro-inflammatory mediators. Transcript expression of vascular MMP2 (a), MMP9 (b), MCP-1 (c) and RANTES (d) assessed by qPCR in WKY and SHRSP rats. Data are expressed as mean ± SEM normalised to 18S (*n* = 6–10 per group). *P < 0.05 vs WKY.

Increased vascular stiffness in hypertensive rats was associated with increased collagen Type I to Type III ratio (Fig. 3), increased vascular fibronectin (Fig. 4a), TGFβ (Fig. 4b) protein expression and p66Shc activation (Fig. 4c). In addition, transcript expression of matrix metalloproteinase (MMP)2 (Fig. 5a) and MMP9 (Fig. 5b) was elevated in hypertensive animals. Expression of pro-inflammatory molecules, RANTES (Fig. 5c) and MCP-1 (Fig. 5d) was higher in vessels from SHRSP versus normotensive WKY rats.

### 3.5 Vascular redox profile is altered in SHRSP rats

**Fig. 6:**
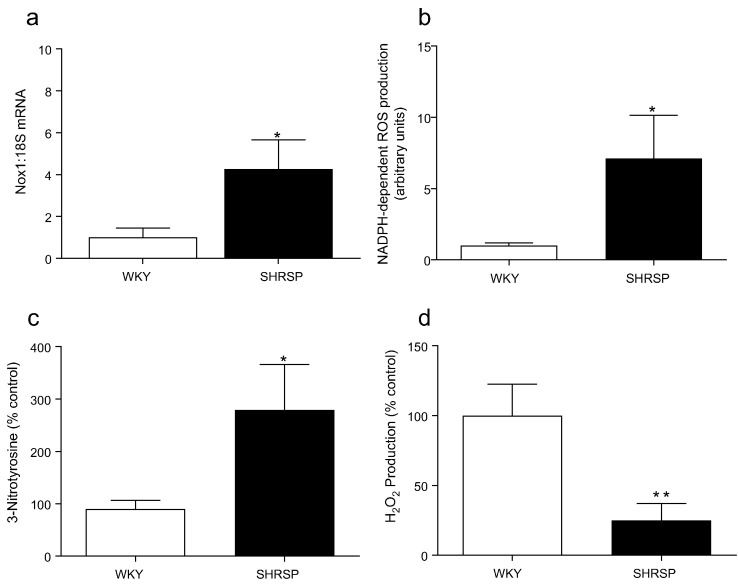
Nox expression and redox status in WKY and SHRSP. Gene expression of vascular Nox1 (a), assessment of vascular NADPH oxidase activity by lucigenin-enhanced chemiluminescence, expressed as relative light units (RLU) per microgram protein (b) nitrotyrosine levels by ELISA assessing 3-Nitrotyrosine production (c) and H_2_O_2_ by Amplex Red assay (d). Bars represent the mean ± SEM (*n* = 5–7 per group), *P < 0.05, **P < 0.01 vs WKY. (For interpretation of the references to colour in this figure legend, the reader is referred to the web version of this article.)

Mesenteric arteries from SHRSP displayed elevated levels of Nox1 transcript level (Fig. 6a). Expression of the other Nox isoforms and subunits was not altered in SHRSP (Supplemental figs. 3a-f). Elevated levels of Nox1 were associated with an increase in NADPH-dependent ROS generation and nitrotyrosine levels (Fig. 6b, c) and decreased levels of H_2_O_2_ (Fig. 6d).

### 3.6 MR blockade normalises pro-inflammatory and pro-fibrotic vascular signaling in SHRSP rats

**Fig. 7:**
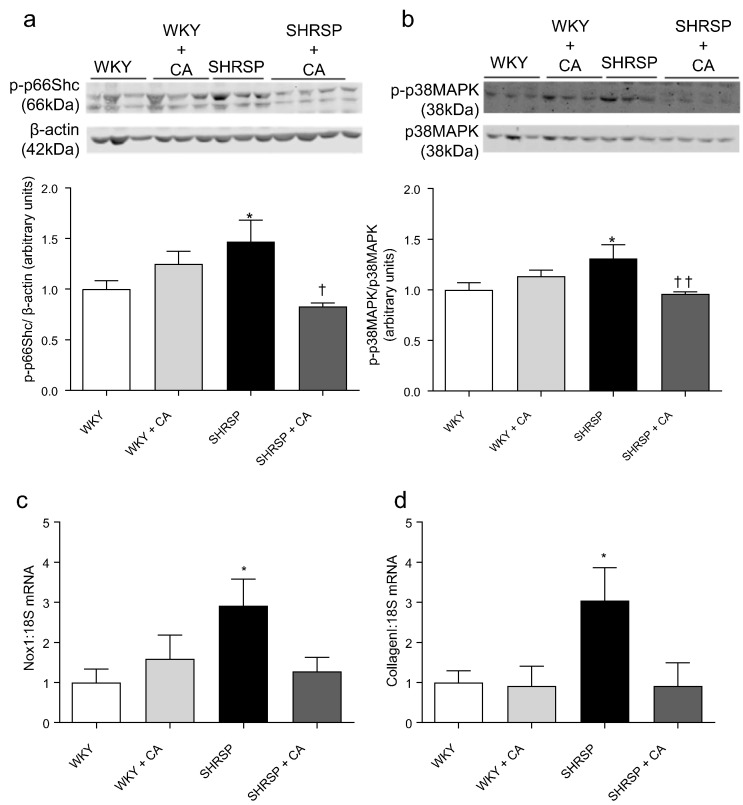
Blockade of mineralocorticoid receptor (MR) reduces pro-fibrotic signaling in aortas of SHRSP rats. Effects of canrenoic acid (CA) (10 mg/kg/day) treatment on vascular activation (phosphorylation) of p66Shc (a) and p38MAPK (b), and Nox1 (c) and fibronectin protein expression (d). Bars represent the mean ± SEM (*n* = 3–4 per group). *P < 0.05 vs WKY vehicle, †*p* < 0.05, ††*P* < 0.01 vs SHRSP vehicle. CA = canrenoic acid.

To determine whether MR blockade in vivo influences the pro-inflammatory and pro-fibrotic vascular phenotype in SHRSP, a group of WKY and SHRSP rats were administered the MR blocker CA (10 mg/kg/day) in drinking water. In aortas of CA-treated SHRSP, activation of pro-inflammatory and pro-fibrotic signaling molecules p66Shc (Fig. 7a) and p38MAPK (Fig. 7b) was reduced and increased expression of Nox1 was prevented (Fig. 7c, Supplemental Fig. 4a). Collagen I mRNA expression was significantly increased in SHRSP, a phenomenon that was prevented by CA treatment (Fig. 7d). CA did not influence fibronectin expression in WKY or SHRSP rats (Supplemental Fig. 4b). Collagen III mRNA level was not altered in SHRSP and unaffected by CA (Supplemental Fig. 4c).

### 3.7 Aldosterone-induced ROS production is Nox1-dependent in VSMCs

**Fig. 8:**
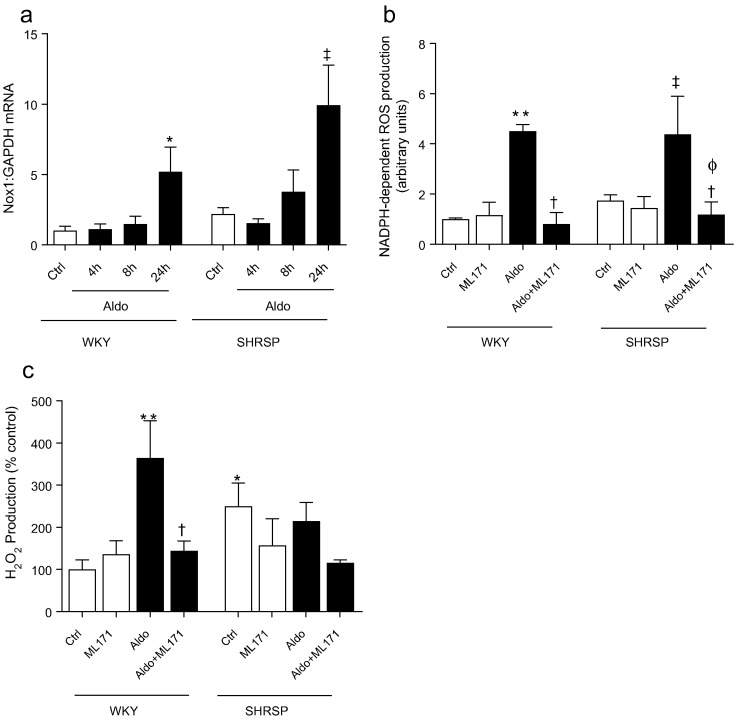
Aldosterone induces Nox1 expression and ROS production in a Nox1-dependent manner in rat VSMCs. Aldosterone (10^− 7^ mol/L) induced expression of Nox1 transcript, relative to GAPDH (a). Aldosterone-induced NADPH-dependent ROS (b) and H_2_O_2_ (c) production is prevented by Nox1 inhibition (ML171) in WKY VSMCs. Bars represent the mean ± SEM (*n* = 4–8 per group). *P < 0.05, **P < 0.001 vs WKY vehicle, †*p* < 0.05 vs Aldosterone stimulated, ‡*P* < 0.01 vs SHRSP vehicle, ΦP < 0.01 vs SHRSP + Aldosterone.

To further investigate the association between aldosterone and Nox1, we examined VSMCs from WKY and SHRSP stimulated with aldosterone. Aldosterone induced an increase in Nox1 expression in WKY, an effect that was amplified in SHRSP VSMCs (Fig. 8a). Aldosterone-induced ROS production in WKY and SHRSP VSMCs was prevented by ML171 (Fig. 8b). ML171 normalised aldosterone-induced H_2_O_2_ production in WKY VSMCs whilst SHRSP VSMCs displayed elevated basal H_2_O_2_ production, which was not enhanced with aldosterone or altered by ML171 (Fig. 8c).

### 3.8 Aldosterone-induced vascular signaling is blunted in Nox1-/y mice

**Fig. 9:**
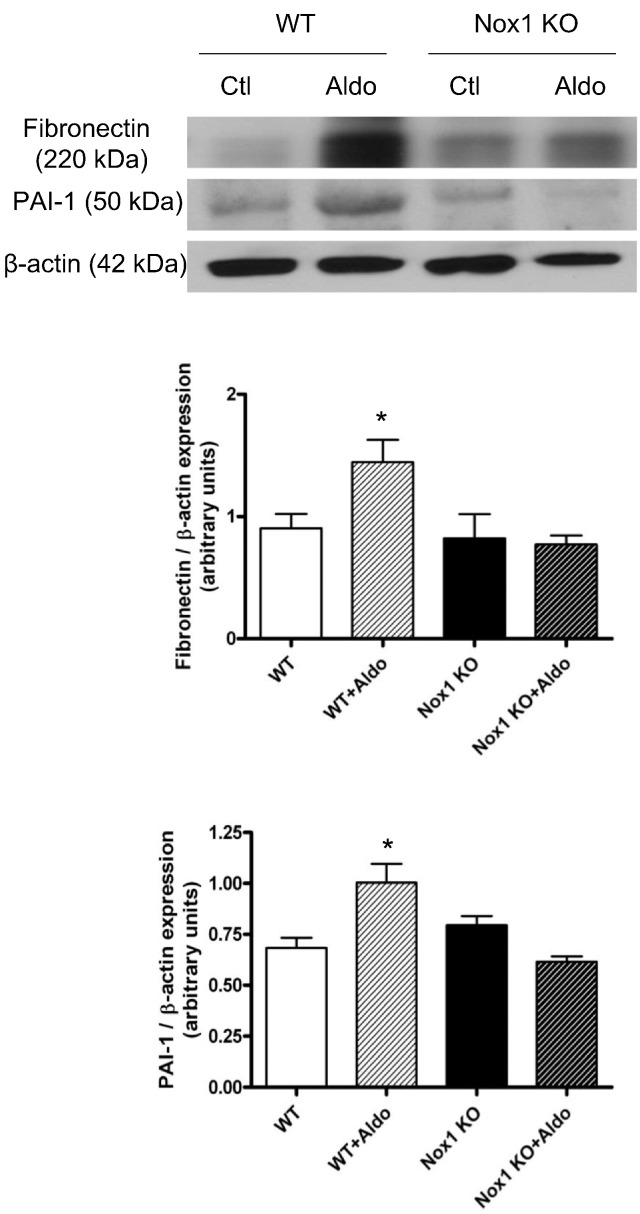
Aldosterone-induced vascular pro-fibrotic signaling is blunted in Nox1 deficient mice. Aldosterone infusion (3 × 10^− 4^ mol/L/kg/day) for 4 weeks increased protein levels of fibrosis markers, fibronectin (a) and PAI-1 (b) in wild-type but not Nox1 deficient mice. Bars represent the mean ± SEM (*n* = 6 per group). *P < 0.05 vs wild-type control.

A role for aldosterone and Nox1 in vascular fibrosis was further evaluated by studying aldosterone-infused wild-type and Nox1-/y mice. Aldosterone stimulated expression of fibronectin and the pro-fibrotic marker PAI-1 in vessels from wild-type but not from Nox1-deficient mice (Fig. 9).

## 4 Discussion

Findings from our study demonstrate that in SHRSP 1) elevated blood pressure is associated with increased levels of plasma aldosterone and galectin-3, vascular stiffness, arterial fibrosis and inflammation, 2) vascular hypercontractility and pro-inflammatory and pro-fibrotic signaling is ameliorated by MR blockade and Nox1 inhibition, and 3) aldosterone-mediated signaling and vascular fibrosis is partially Nox1-dependent. Together our data suggest a role for aldosterone/MR-mediated Nox1 activation in vascular damage and dysfunction in SHRSP. We also identify a novel signaling pathway involving p66Shc through which aldosterone-MR may influence vascular fibrosis.

SHRSP rats are characterised by severe hypertension and associated endothelial dysfunction and vascular damage. Exact molecular mechanisms contributing to these processes remain unclear but aldosterone may play a role. Previous studies demonstrated that plasma aldosterone is increased in SHRSP [24], similar to what we observed in our study. Moreover, MR blockers reduce vascular damage without effects on blood pressure in SHRSP on normal diet [19], [25] and as demonstrated in our study, MR blockade improved vascular dysfunction and ameliorated pro-inflammatory and profibrotic signaling in SHRSP. Our findings are in line with earlier studies, which showed antifibrotic effects of MR antagonists in SHRSP [19], [25].

Aldosterone induces fibrosis, which may contribute to vascular remodeling in hypertension. Increasing evidence indicates a role for galectin-3 in cardiovascular fibrosis and cardiac dysfunction and levels of this biomarker are now being used clinically as a predictor of heart failure [26]. In the cardiovascular system galectin-3 has been implicated in the development of fibrosis where galectin-3 infusion increases cardiac collagen type I:type III ratio in rats [27] and recombinant galectin-3 induces fibroblast proliferation and collagen synthesis in vitro. The importance of galectin-3 in the development of aldosterone-mediated fibrosis has been highlighted by the observation that mice lacking galectin-3 are protected against aldosterone-induced elevation in collagen expression in the heart [28] and aorta [4]. In rat VSMCs, over expression of galectin-3 enhanced aldosterone-induced collagen 1 synthesis, whereas spironolactone or modified citrus pectin (galectin-3 inhibitor) reversed these effects [4]. It may be possible that aldosterone stimulates production of galectin-3, since increased plasma levels of galectin-3 in patients with primary hyperaldosteronism were normalised following adrenalectomy [29]. In our study, plasma levels of galectin-3 were closely associated with hyperaldosteronism and vascular dysfunction and fibrosis. At the molecular level, increased expression of vascular collagen, fibronectin, TGFβ, MMPs, RANTES and MCP-1, together with activation of p66Shc in SHRSP rats, supports the pro-fibrotic and pro-inflammatory vascular phenotype in hypertension.

To further support a profibrotic role of aldosterone-MR in SHRSP, we previously showed that MR blockade with eplerenone prevents vascular remodeling and cardiac fibrosis [19]. Others demonstrated that spironolactone attenuates vascular fibrosis in cerebral arteries in SHRSP [25]. Our results here support these previous studies, since increased mRNA expression of collagen I in SHRSP, was normalised in mice treated with MRA. Of note, although collagen I expression was increased at the gene level, it was not significantly increased at the protein level and may relate, at least in in part, to possible degradation by MMPs. Supporting this notion are our findings that vascular mRNA expression of MMP2 and MMP9 was increased in SHRSP. Similar MMP findings have been observed by others in SHRSP [30]. Although CA prevented an increase in collagen expression in SHRSP, it did not influence fibronectin expression. Reasons for this may relate to the relatively low dose of MRA used. We treated rats with 10 mg/kg/day, whereas other studies used MR blockers at higher doses [19], [25]. Perhaps a higher dose would have had greater antifibrotic effects in our model.

SHRSP rats had elevated levels of phosphorylated p66Shc, an important redox-sensitive signaling molecule that has been implicated in cardiovascular aging, vascular injury and renal dysfunction [31], [32], [33], [34]. MR blockade prevented activation of vascular p66Shc in SHRSP, indicating an important role for p66Shc in aldosterone-MR signaling in vascular tissue. To our knowledge these are the first studies suggesting that aldosterone signals through p66Shc in vascular cells and support those in kidney cells, where aldosterone-induced epithelial-to-mesenchymal transition in renal proximal tubular cells was found to involve p66Shc [34]. Our findings unravel a potential new signaling pathway through which aldosterone-MR signals.

Molecular mechanisms underlying the vascular phenotype in SHRSP and the role of aldosterone in these processes remain elusive, but oxidative stress may be important. Aldosterone influences redox-sensitive signaling in vascular smooth muscle cells and stimulates Nox1 to induce generation of ROS [16]. In our study, expression of vascular Nox1, but not other Nox isoforms or Nox subunits, was increased. Inhibition of Nox1 and its regulator Rac1/2 with ML171 and EHT1864 respectively, restored hypercontractility suggesting a functional link between Nox1 upregulation and impaired vascular function in hypertension. Because of the vasoprotective effects of eplerenone and ML171 in SHRSP, we questioned whether aldosterone actions may be mediated through Nox1-dependent processes in this model.

Increased vascular expression of Nox1 was associated with increased levels of vascular O_2_^•-^ and ONOO^−,^ which may contribute to oxidative stress-induced vascular injury. Whereas generation of O_2_^•−^ and ONOO^−^ was increased in SHRSP, levels of vascular H_2_O_2_ were reduced. Since H_2_O_2_ may act as an endothelium-derived relaxing factor_,_ decreased bioavailability of this ROS may lead to reduced vasorelaxation and increased contraction [35], [36]. To explore the putative relationship between aldosterone and Nox1 in SHRSP we studied cultured VSMCs from SHRSP and WKY. Aldosterone induced expression of Nox1 in WKY with an amplified effect in SHRSP. Nox1 inhibition attenuated aldosterone-stimulated ROS generation in VSMCs from both normotensive and hypertensive rats. In contrast to findings in intact arteries, SHRSP VSMCs produced more H_2_O_2_ than WKY VSMCs. Reasons for this may relate to the fact that H_2_O_2_ production is differentially regulated with variable functional effects in endothelial cells and in VSMCs. In particular, in endothelial cells, H_2_O_2_ acts as a vasodilator, whereas in VSMCs H_2_O_2_ may promote vasoconstriction [35], [36]. Hence the divergent findings in our study between isolated VSMC versus intact arteries likely reflects the contribution of endothelial cells in whole vessels. Our findings that ML171 inhibits aldosterone effects, suggest a role for Nox1 in aldosterone-mediated vascular actions. The observation that ML171 reduced NADPH-dependent ROS but not H_2_O_2_ production in SHRSP VSMCs may suggest that the elevated H_2_O_2_ levels in these cells are Nox1-independent.

To further elucidate the relationship between aldosterone, Nox and vascular fibrosis, in vivo, aldosterone-infused Nox1-deficient mice were studied. Whereas vascular content of fibronectin and PAI-1 was increased in aldosterone-infused wild-type mice, this was not evident in mice lacking Nox1. These findings suggest an important role for Nox1 in aldosterone-mediated vascular changes and support our findings in SHRSP and cultured VSMCs.

## 5 Conclusions

Aldosterone and oxidative stress may be important regulators of vascular changes associated with hypertension. Our data suggest that aldosterone/MR-induced vascular dysfunction and arterial remodeling involve Nox1-mediated redox-p66Shc signaling. We define a novel molecular mechanism underlying vascular changes in hypertension. Targeting MR and Nox1 may have vasoprotective effects.

## Appendix A Supplementary data

Supplementary figures. Image 1

## References

[1] McMaster W.G., Kirabo A., Madhur M.S., Harrison D.G. (2015). Inflammation, immunity, and hypertensive end-organ damage. Circ. Res..

[2] Harvey A., Montezano A.C., Lopes R.A., Rios F., Touyz R.M. (2016). Vascular fibrosis in aging and hypertension: molecular mechanisms and clinical implications. Can. J. Cardiol..

[3] Park J.B., Schiffrin E.L. (2002). Cardiac and vascular fibrosis and hypertrophy in aldosterone-infused rats: role of endothelin-1. Am. J. Hypertens..

[4] Calvier L., Miana M., Reboul P., Cachofeiro V., Martinez-Martinez E., de Boer R.A. (2013). Galectin-3 mediates aldosterone-induced vascular fibrosis. Arterioscler. Thromb. Vasc. Biol..

[5] Zannad F., McMurray J.J., Krum H., van Veldhuisen D.J., Swedberg K., Shi H. (2011). Eplerenone in patients with systolic heart failure and mild symptoms. N. Engl. J. Med..

[6] Tarjus A., Belozertseva E., Louis H., El Moghrabi S., Labat C., Lacolley P. (2015). Role of smooth muscle cell mineralocorticoid receptor in vascular tone. Pflügers Archiv..

[7] Montezano A.C., Tsiropoulou S., Dulak-Lis M., Harvey A., Camargo Lde L., Touyz R.M. (2015). Redox signaling, Nox5 and vascular remodeling in hypertension. Curr. Opin. Nephrol. Hypertens..

[8] Cowley A.W., Yang C., Zheleznova N.N., Staruschenko A., Kurth T., Rein L. (2015). Evidence of the importance of Nox4 in production of hypertension in dahl salt-sensitive rats. Hypertension.

[9] Montezano A.C., Touyz R.M. (2012). Molecular mechanisms of hypertension—reactive oxygen species and antioxidants: a basic science update for the clinician. Can. J. Cardiol..

[10] Gray S.P., Di Marco E., Okabe J., Szyndralewiez C., Heitz F., Montezano A.C. (2013). NADPH oxidase 1 plays a key role in diabetes mellitus-accelerated atherosclerosis. Circulation.

[11] Schroder K., Zhang M., Benkhoff S., Mieth A., Pliquett R., Kosowski J. (2012). Nox4 is a protective reactive oxygen species generating vascular NADPH oxidase. Circ. Res..

[12] Ray R., Murdoch C.E., Wang M., Santos C.X., Zhang M., Alom-Ruiz S. (2011). Endothelial Nox4 NADPH oxidase enhances vasodilatation and reduces blood pressure in vivo. Arterioscler. Thromb. Vasc. Biol..

[13] Basuroy S., Bhattacharya S., Leffler C.W., Parfenova H. (2009). Nox4 NADPH oxidase mediates oxidative stress and apoptosis caused by TNF-alpha in cerebral vascular endothelial cells. Am. J. Physiol. Cell. Physiol..

[14] Yogi A., Mercure C., Touyz J., Callera G.E., Montezano A.C., Aranha A.B. (2008). Renal redox-sensitive signaling, but not blood pressure, is attenuated by Nox1 knockout in angiotensin II-dependent chronic hypertension. Hypertension.

[15] Shi G., Fu Y., Jiang W., Yin A., Feng M., Wu Y. (2011). Activation of Src-ATF1 pathway is involved in upregulation of Nox1, a catalytic subunit of NADPH oxidase, by aldosterone. Endocr. J..

[16] Wei H., Mi X., Ji L., Yang L., Xia Q., Wei Y. (2010). Protein kinase C-δ is involved in induction of NOX1 gene expression by aldosterone in rat vascular smooth muscle cells. Biochemistry.

[17] Fu Y., Shi G., Wu Y., Kawai Y., Tian Q., Yue L. (2011). The mitochondria mediate the induction of NOX1 gene expression by aldosterone in an ATF-1-dependent manner. Cell. Mol. Biol. Lett..

[18] Akasaki T., Ohya Y., Kuroda J., Eto K., Abe I., Sumimoto H., Iida M. (2006). Increased expression of gp91phox homologues of NAD (P) H oxidase in the aortic media during chronic hypertension: involvement of the renin-angiotensin system. Hypertens. Res..

[19] Endemann D.H., Touyz R.M., Iglarz M., Savoia C., Schiffrin E.L. (2004). Eplerenone prevents salt-induced vascular remodeling and cardiac fibrosis in stroke-prone spontaneously hypertensive rats. Hypertension.

[20] Kilkenny C., Browne W., Cuthill I.C., Emerson M., Altman D.G. (2010). Animal research: reporting in vivo experiments: the ARRIVE guidelines. Br. J. Pharmacol..

[21] Lopes R.A., Neves K.B., Tostes R.C., Montezano A.C., Touyz R.M. (2015). Downregulation of nuclear factor erythroid 2-related factor and associated antioxidant genes contributes to redox-sensitive vascular dysfunction in hypertension. Hypertension.

[22] Rehman A., Leibowitz A., Yamamoto N., Rautureau Y., Paradis P., Schiffrin E.L. (2012). Angiotensin type 2 receptor agonist compound 21 reduces vascular injury and myocardial fibrosis in stroke-prone spontaneously hypertensive rats. Hypertension.

[23] Ibrahim J., McGee A., Graham D., McGrath J.C., Dominiczak A.F. (2006). Sex-specific differences in cerebral arterial myogenic tone in hypertensive and normotensive rats. Am. J. Physiol. Heart Circ. Physiol..

[24] Sampson A.K., Mohammed D., Beattie W., Graham D., Kenyon C.J., Al-Dujaili E.A. (2014). Introgressed chromosome 2 quantitative trait loci restores aldosterone regulation and reduces response to salt in the stroke-prone spontaneously hypertensive rat. J. Hypertens..

[25] Rigsby C.S., Ergul A., Portik Dobos V., Pollock D.M., Dorrance A.M. (2011). Effects of spironolactone on cerebral vessel structure in rats with sustained hypertension. Am. J. Hypertens..

[26] Christenson R.H. (2015). Evolving role of galectin-3 as a cardiac biomarker: heart failure with preserved ejection fraction and renal function, important pieces of the puzzle. JACC Heart Fail..

[27] Sharma U.C., Pokharel S., van Brakel T.J., van Berlo J.H., Cleutjens J.P., Schroen B. (2004). Galectin-3 marks activated macrophages in failure-prone hypertrophied hearts and contributes to cardiac dysfunction. Circulation.

[28] Martínez-Martínez E., Calvier L., Fernández-Celis A., Rousseau E., Jurado-López R., Rossoni L.V. (2015). Galectin-3 blockade inhibits cardiac inflammation and fibrosis in experimental hyperaldosteronism and hypertension. Hypertension.

[29] Liao C.W., Lin Y.T., Wu X.M., Chang Y.Y., Hung C.S., Wu V.C., Wu K.D., Lin Y.H., TAIPAI Study Group (2016). The relation among aldosterone, galectin-3, and myocardial fibrosis: a prospective clinical pilot follow-up study. J. Investig. Med..

[30] Ueno M., Wu B., Nishiyama A., Huang C.L., Hosomi N., Kusaka T., Nakagawa T., Onodera M., Kido M., Sakamoto H. (2009). The expression of matrix metalloproteinase-13 is increased in vessels with blood-brain barrier impairment in a stroke-prone hypertensive model. Hypertens. Res..

[31] Migliaccio E., Giorgio M., Mele S., Pelicci G., Reboldi P., Pandolfi P.P. (1999). The p66shc adaptor protein controls oxidative stress response and life span in mammals. Nature.

[32] Francia P., delli Gatti C., Bachschmid M., Martin-Padura I., Savoia C., Migliaccio E. (2004). Deletion of p66shc gene protects against age-related endothelial dysfunction. Circulation.

[33] Lebiedzinska M., Duszynski J., Rizzuto R., Pinton P., Wieckowski M. (2009). Age-related changes in levels of p66Shc and serine 36-phosphorylated p66Shc in organs and mouse tissues. Arch. Biochem. Biophys..

[34] Yuan Y., Chen Y., Zhang P., Huang S., Zhu C., Ding G. (2012). Mitochondrial dysfunction accounts for aldosterone-induced epithelial-to-mesenchymal transition of renal proximal tubular epithelial cells. Free Radic. Biol. Med..

[35] Matoba T., Shimokawa H., Nakashima M., Hirakawa Y., Mukai Y., Hirano K. (2000). Hydrogen peroxide is an endothelium-derived hyperpolarizing factor in mice. J. Clin. Invest..

[36] Burke-Wolin T., Abate C.J., Wolin M.S., Gurtner G.H. (1991). Hydrogen peroxide-induced pulmonary vasodilation: role of guanosine 3′,5′-cyclic monophosphate. Am. J. Phys..

