# Supplementary material for: Vascular dysfunction and fibrosis in stroke-prone spontaneously hypertensive rats: The aldosterone-mineralocorticoid receptor-Nox1 axis
Source: Life Sci. 2017 Jun 15;179:110–9. doi: 10.1016/j.lfs.2017.05.002 (PMC5446265; doi:10.1016/j.lfs.2017.05.002)

Supplemental Text

Vascular Dysfunction and Fibrosis in Stroke-Prone Spontaneously Hypertensive Rats : The Aldosterone-mineralocorticoid receptor-Nox1 Axis.

Adam P HARVEY[a], Augusto C MONTEZANO[a], Katie Y HOOD[a], Rheure A LOPES[a], Francisco RIOS[a], Graziela CERAVOLO[b], Delyth GRAHAM[a], Rhian M TOUYZ[a].

a Institute of Cardiovascular and Medical Sciences, University of Glasgow, United Kingdom.

b Department of Physiological Sciences, State University of Londrina, Londrina, Brazil.

To whom correspondence should be addressed:

Rhian M Touyz MD, PhD

Institute of Cardiovascular and Medical Sciences,

University of Glasgow,

126 University Place, Glasgow, G12 8TA,

Phone: + 44 (0)141 330 7775/7774, Fax: + 44 (0)141 330 3360,

Email: Rhian.Touyz@glasgow.ac.uk

Figure legends

Supplemental figure 1.

Effects of hypertension on endothelium independent relaxation. Assessment of vascular relaxation to SNP in rat mesenteric arteries by wire myography. Curves represent the mean±SEM (n=5–15).

Supplemental figure 2.

Effects of hypertension on vascular structure. Vascular structure external diameter (a), Internal diameter (b), Wall thickness (c) and Cross Sectional Area (CSA) (d) assessed by pressure myography in normotensive and hypertensive rats. Curves represent the mean±SEM (n=5–15).

Supplemental figure 3.

Gene expression of vascular Nox isoforms and NADPH oxidase subunits. Vascular transcript levels of Nox2 (a), Nox4 (b), p22phox (c), p47phox (d), NoxA1 (e), NoxO1 (f), assessed by qPCR in WKY and SHRSP rats. Data are expressed as mean±SEM, where mRNA levels are normalised to 18S (n=5–10 per group). *P<0.05 vs WKY.

Supplemental figure 4.

Effects of canrenoic acid (CA) (10mg/kg/day) treatment on vascular Nox1 (a) and fibronectin protein expression (b) and collagen III mRNA expression (c). Data are expressed as mean±SEM; protein expression is normalised to β-Actin and mRNA levels are normalised to β-Actin (n=3–4 per group). *P<0.05 vs WKY vehicle. CA=canrenoic acid.

Supplemental figure 1

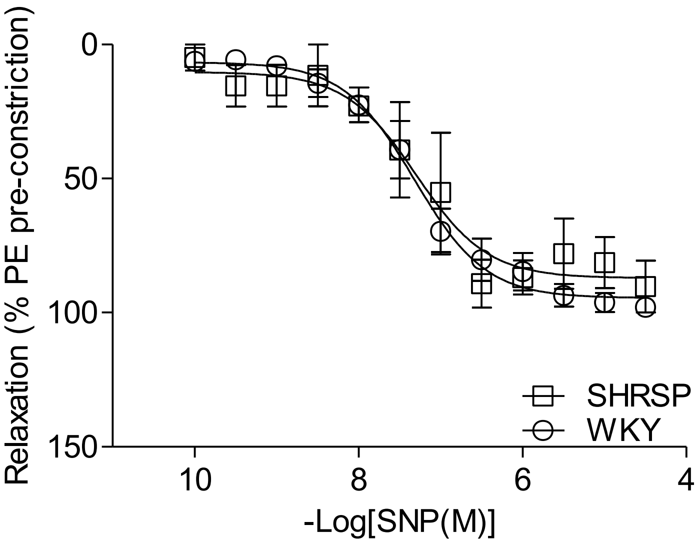

Supplemental figure 2

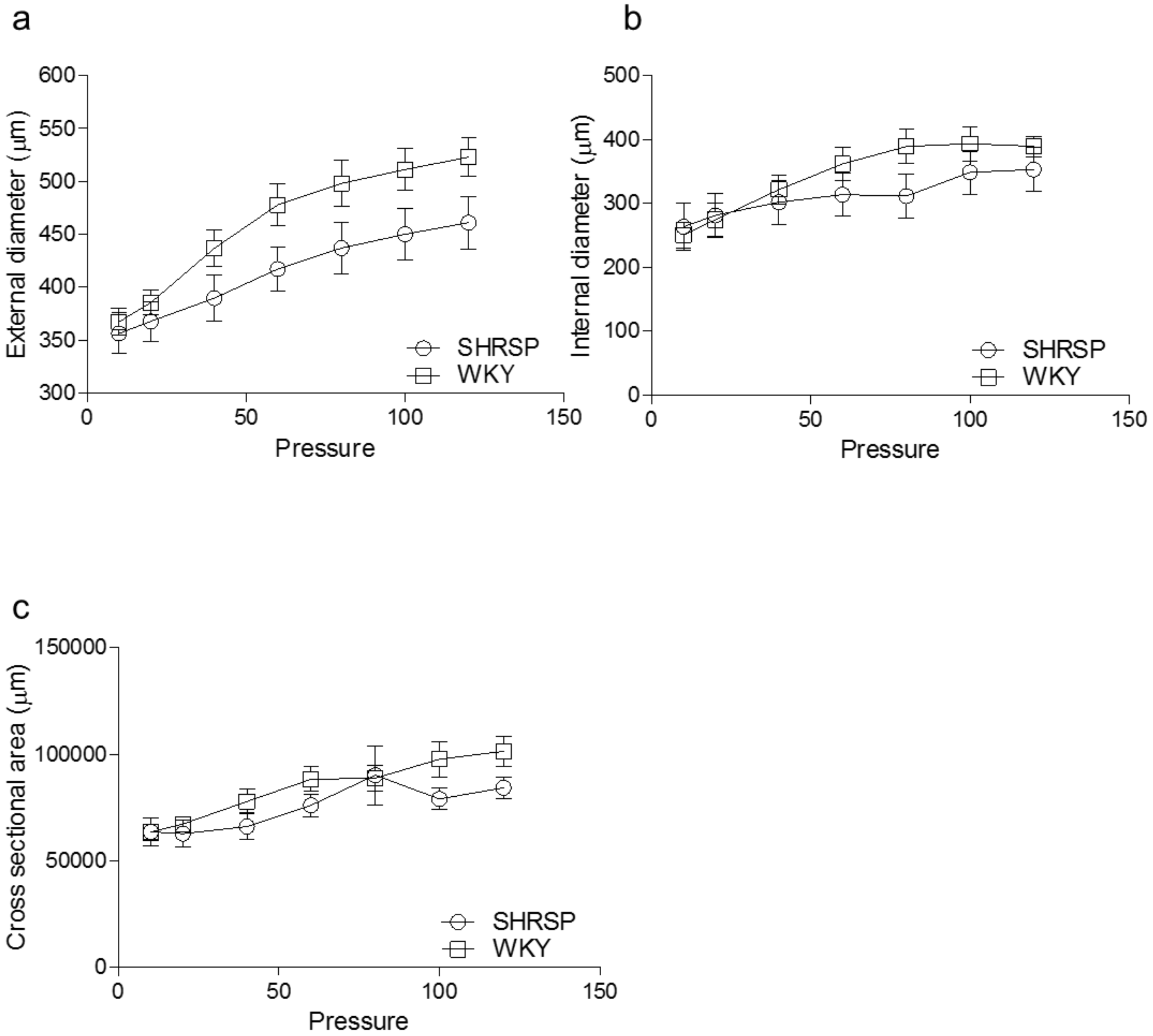

Supplemental figure 3

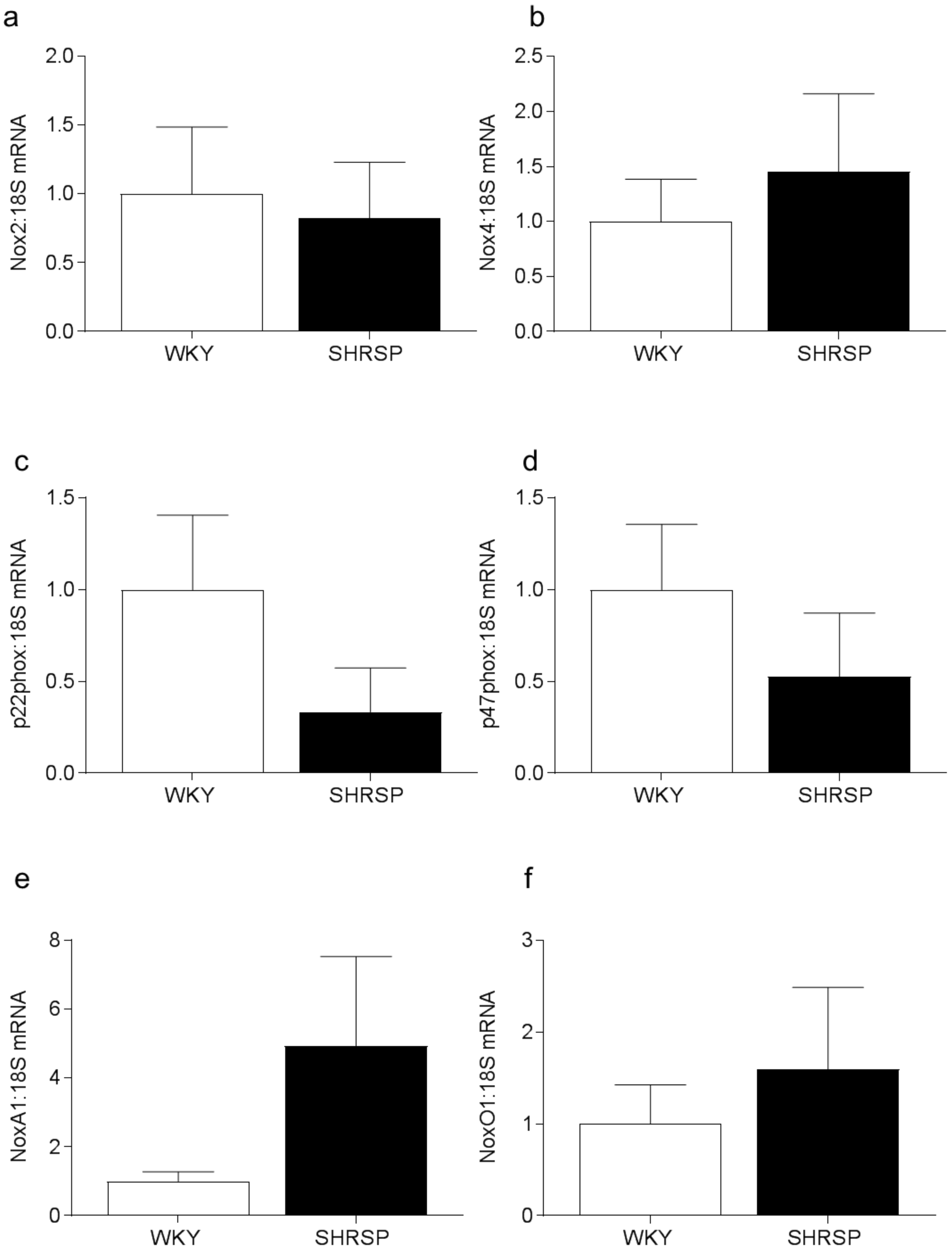

Supplemental figure 4

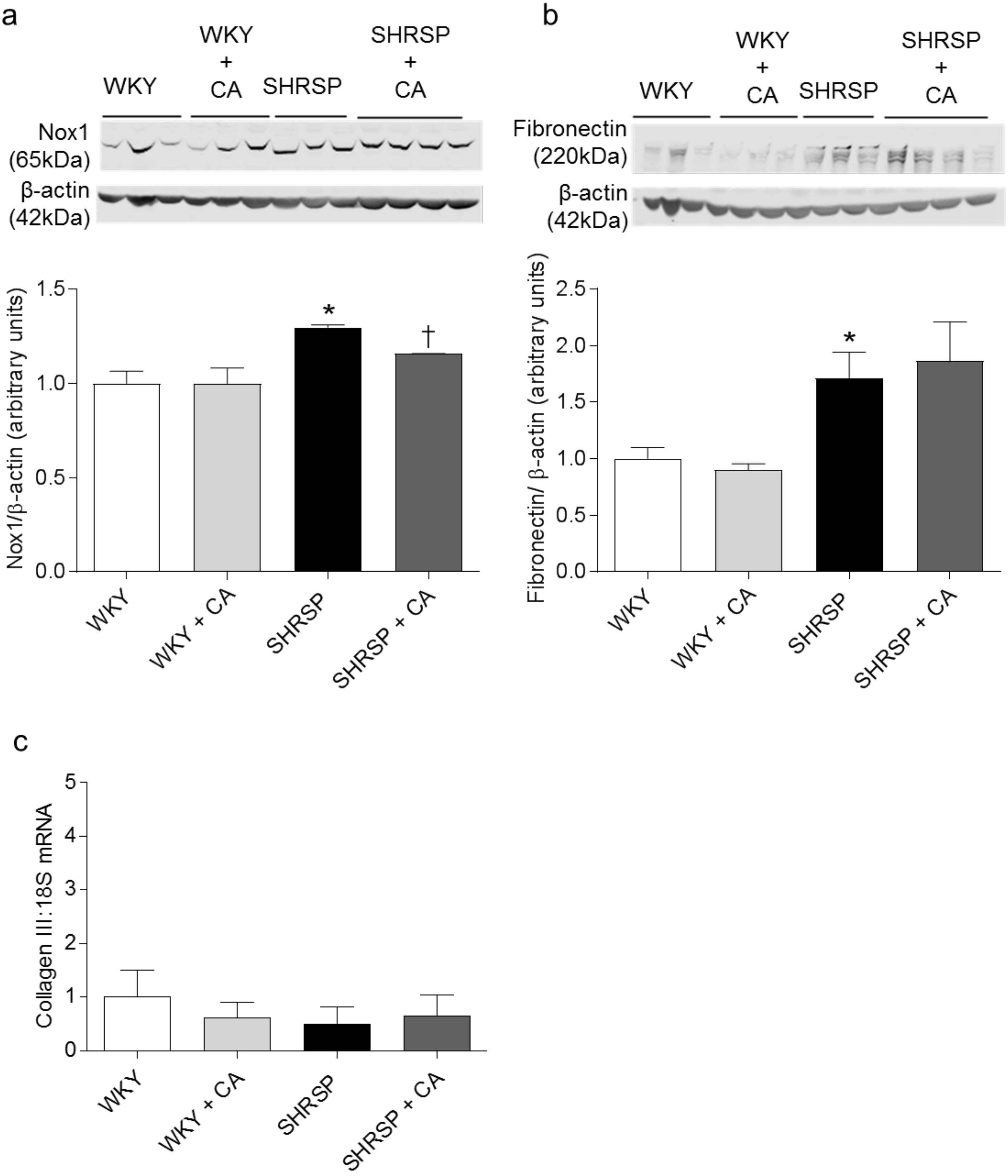

Supplement: Supplementary file 1 — Supplementary figures. [file mmc1.pdf]
